# Evaluation of internet access and utilization by medical students in Lahore, Pakistan

**DOI:** 10.1186/1472-6947-11-37

**Published:** 2011-05-30

**Authors:** Nauman A Jadoon, Muhammad F Zahid, Hafiz Mansoorulhaq, Sami Ullah, Bilal A Jadoon, Ali Raza, Mansoor Hussain, Rehan Yaqoob, Mohammad A Shahzad

**Affiliations:** 1 Department of Medicine, Nishtar Medical College Hospital, Multan, Pakistan; 2 Center for Biomedical Research, Lahore, Pakistan; 3 Department of Surgery, Fatima Memorial College of Medicine and Dentistry, Lahore, Pakistan; 4 Department of Surgery, Sharif Medical and Dental College, Lahore, Pakistan; 5 Services Institute of Medical Sciences, Lahore, Pakistan; 6 Institute of Management Sciences, Peshawar, Pakistan; 7 Postgraduate Medical Institute, Lahore, Pakistan

## Abstract

**Background:**

The internet is increasingly being used worldwide in imparting medical education and improving its delivery. It has become an important tool for healthcare professionals training but the data on its use by medical students in developing countries is lacking with no study on the subject from Pakistan. This study was, therefore, carried out with an aim to evaluate the pattern of internet access and utilization by medical students in Pakistan.

**Methods:**

A structured pre-tested questionnaire was administered to a group of 750 medical students in clinical years studying at various public and private medical colleges in Lahore. The questions were related to patterns of internet access, purpose of use and self reported confidence in performing various internet related tasks, use of health related websites to supplement learning and the problems faced by students in using internet at the institution.

**Results:**

A total of 532 medical students (70.9%) returned the questionnaire. The mean age of study participants was 21.04 years (SD 1.96 years). Majority of the respondents (84.0%) reported experience with internet use. About half of the students (42.1%) were using internet occasionally with 23.1%, 20.9% and 13.9% doing so frequently, regularly and rarely respectively. About two third of the students (61.0%) stated that they use internet for both academic and professional activities. Most of the participants preferred to use internet at home (70.5%). Self reported ability to search for required article from PubMed and PakMedinet was reported by only 34.0% of the entire sample. Students were moderately confident in performing various internet related tasks including downloading medical books from internet, searching internet for classification of diseases and downloading full text article. Health related websites were being accessed by 55.1% students to supplement their learning process. Lack of time, inadequate number of available computers and lack of support from staff were cited as the most common problems faced by students while accessing internet in the institution premises. There were significant differences among male and female students with respect to the place of internet use (p = 0.001) and the ability to search online databases for required articles (p = 0.014).

**Conclusions:**

Majority of the medical students in this study had access to internet and were using it for both academic and personal reasons. Nevertheless, it was seen that there is under utilization of the potential of internet resources to augment learning. Increase in awareness, availability of requisite facilities and training in computing skills are required to enable better utilization of digital resources of digital resources by medical students.

## Background

The development and evolution of internet has brought profound changes in the health care delivery systems across the globe ranging from education and training to diagnosis and patient management [1]. Internet has fundamentally transformed the patient management practices of health care professionals. According to an estimate, 30% of a physician time will be spent in the use of various information and communication technology tools in 2010 [2]. Internet is a cost effective medium of communication which can help in meeting the complex information needs of healthcare professionals and has important implications in medical education. It can serve as an important learning tool in medical education by providing access to latest evidence anytime and anywhere. It is especially useful for students from developing countries helping them to keep abreast of ever expanding knowledge bridging the gap resulting from scarcity of resources.

Internet has a number of applications in the field of medicine and health. It provides instant access to relevant up to date information at the point of care making it easy for the health care providers to practice evidence based medicine. It offers clinician the ability to manage patients in remote areas while being away from them and makes it possible to interact with colleagues on clinical issue via videoconferencing. It also promotes research and learning by providing access to medical and other online databases [3-5]. It is highly imperative that health care professionals are able to fully utilize the available resources on internet so that they can evaluate the large quantity of medical literature available and find the requisite information quickly.

The internet is increasingly being used worldwide in imparting medical education and improving its delivery. Internet finds many uses in the medical domain including but not limited to rapid seamless delivery of educational material, access to online databases, exploring the application of theoretical ideas and use of various interactive tools to enhance understanding of complex ideas [6-8]. In addition the beauty of internet lies in the fact that it allows learners the convenience of studying at their own pace without the hassle of attending classes at a fixed schedule and at a defined place. However, internet mediated learning is not without its share of disadvantages and limitations. These include the lack of infrastructure and limited access in developing countries. Moreover, it is difficult for a busy clinician to find time to keep up to date on latest breakthroughs in medicine. In addition the reluctance of health care professionals to use internet instead of traditional information sources also hinders their ability to fully utilize this information source [9].

Pakistan is the most populous country in the Eastern Mediterranean Region (EMR) of the World Health Organization (WHO). The number of internet users in the country have increased from 133,900 in the year 2000 to 18.5 million in 2010 registering a record growth of 13,716.3% from 2000 to 2010 [10]. Pakistan is included in the list of low internet penetration countries having internet penetration lower than the world average of 23.8% [11]. Currently, the internet population penetration stands at 10.4% representing 2.2% of the users in Asia as of June 2010 [10]. The country ranks 10th in the EMR, 14th in the lower middle income countries and 87th globally in the 2010 network readiness index released by the world economic forum [12].

The various advantages of modern information technology tools can only be materialized in the developing countries if the health care professionals are well versed and adequately trained in basic computing skills. Such training requires a survey to access the baseline proficiency and utilization pattern to identify the areas needing attention which is main aim of this survey. Although the students are provided basic training on use of internet and searching medical databases and have a research project as compulsory part of coursework involving writing a scientific article, it is not known how this training helps them in acquiring the skills necessary to undertake independent online learning. In addition, there is limited data available on the use of internet by health care professionals in developing countries. Furthermore, there is no study on the access and utilization pattern of medical students in Pakistan to the best of our knowledge. The study is also needed to aid in developing evidence base for improving usage of online resources and their integration into education and training to bridge the information gap resulting from scarcity of resources. This study was, therefore, conducted with an aim to determine the patterns of internet access and utilization among medical students undergoing clinical training. The study also assessed the self rated ability of students to search online databases, self reported confidence in performing various internet related tasks, use of health related websites to supplement learning and distribution of problems faced by students in assessing the information at their institution.

## Methods

The study was carried out at four medical colleges in Lahore which were randomly selected after dividing the city into two parts. Two public and two private medical colleges were selected, one public and private college being from each part of the city. The public medical colleges included Allama Iqbal medical College (AIMC) and Services Institute of Medical Sciences (SIMS). Sharif Medical and Dental College (SMDC) and Fatima Memorial Hospital College of Medicine and Dentistry (FMH) were the private medical colleges included in the study. All the medical schools in Lahore are affiliated with the University of Health Sciences, Lahore (UHS) as are most other public and private medical institutions in the Punjab province, the most populous province of the country. Computer resource labs were established at all the medical colleges affiliated with UHS with funding from the Higher Education Commission (HEC), Pakistan in 2007 [13]. The labs have computers, printers and scanners providing facilities of internet, copying, scanning, printing, access to various online databases through HEC digital library and training to students and faculty of colleges and attached hospitals.

The study population in this study consisted of 750 randomly selected medical students in the clinical years of training (3rd, 4th and Final year of M.B.B.S.). A structured questionnaire designed by the authors was used for the study. It was pretested on a group of students before starting the study. The questionnaire elicited information about demographic profile of students and pattern of internet access and utilization. The questionnaire also assessed self reported confidence of students in performing various internet related tasks such as downloading medical books from internet, searching internet for classification of diseases and retrieval of full text articles. The answers were scored on Likert scale ranging from 1 to 5 with 1 denoting not confident and 5 representing very confident. The responses were used to generate self efficacy scores of students in performing these tasks. Questions about use of health related websites in learning process and the problems faced by students in using internet at campus were also included in the questionnaire. The questionnaire was anonymous to increase participation and reduce the potential of respondent bias. The study was approved by Ethics Review Committee of Nishtar Medical College Hospital Multan. Approval was also sought from each of the institutions before carrying out the study. The forms were distributed among the students after taking informed consent from participants.

Data analysis was carried out using SPSS version 16. Descriptive analyses were performed for various variables. Pearson chi-square test was employed to determine the difference in internet access and utilization and the use of health related websites by gender. A p value of < 0.05 was considered statistically significant.

## Results

A response rate of 70.9% was achieved with a total of 532 students returning filled forms out of 750. The mean age of students was 21.04 years (SD = 1.96 years). Majority of the students were female (59.6%) and studying in fourth year MBBS (36.8%). The response rates were 71.2%, 75.0%, 73.0% and 75.0% for AIMC, FMH, SMDC and SIMS respectively. The demographic profile of the student is presented in Table 1.

**Table 1:** Demographic Profile of the Students

Variable	n(%)*
Age	21.04 (1.96)

Gender	

Male	215 (40.41%)

Female	317 (59.59%)

Institution	

Allama Iqbal Medical College	178 (33.5%)

FMH College of Medicine & Dentistry	75 (14.1%)

Sharif Medical & Dental College	129 (24.2%)

Services Institute of Medical Sciences	150 (28.2%)

Year of Study	

3^rd ^Year MBBS	151 (28.4%)

4^th ^Year MBBS	196 (36.8%)

Final Year MBBS	185 (34.7%)

* Data are mean ± SD for Age

Students' pattern of internet access and use is described in Table 2. Overall 84.0% of the entire sample had experience with internet use. The student surveyed were using internet with various frequencies. Most of the students (42.1%) reported occasional use of internet. Majority of the respondents (61.0%) were using internet for both personal and academic services. Students preferred using internet at home. Google was the most popular search engine and was being used by 88.9% of the participants. Self reported ability to search online databases was documented by only a minority of medical students. When the data of internet access and usage was analyzed according to gender, a statistically significant difference was observed with respect to place of internet use and the ability to search online databases for articles.

**Table 2:** Pattern Internet Access and Use

Item	Total		Male		Female		P Value
		
	n	%	n	%	n	%	
1. Experience with internet use							.156

Yes	440	84.0%	173	81.2%	267	85.9%	

No	84	16.0%	40	18.8%	44	14.1%	

2. Frequency of internet use							.192

Frequently	120	23.1%	54	25.8%	66	21.3%	

Regularly	108	20.9%	50	23.9%	58	18.8%	

Occasionally	218	42.1%	79	37.8%	139	45.0%	

Rarely	72	13.9%	26	12.5%	46	14.9%	

3. Internet services used by students							.219

Personal	108	22.0%	43	21.7%	65	22.1%	

Academic	84	17.0%	27	13.6%	57	19.4%	

Both	300	61.0%	128	64.7%	172	58.5%	

4. Place of internet use							.000

Café	34	6.6%	23	11.1%	11	3.6%	

Home	363	70.5%	121	58.5%	242	78.6%	

Library	107	20.8%	58	28.0%	49	15.9%	

Other	11	2.1%	5	2.4%	6	1.9%	

5. Search engines used							.526

Google	450	88.9%	187	89.9%	263	88.3%	

Yahoo	45	8.9%	15	7.2%	30	10.1%	

Others	11	2.2%	6	2.9%	5	1.6%	

6. Ability to search Pubmed/pakmedinet for article							.014

Yes	166	34.0%	82	40.2%	84	29.5%	

No	323	66.0%	122	59.8%	201	70.5%	

Analysis of students' perceived confidence in performing internet related tasks is shown in Table 3. The results are expressed as perceived self efficacy scores (range 1-5). The results show that students have moderate levels of confidence in performing various internet related tasks. Health related websites were being used by 55.1% of the participants (Table 4). Lack of time to use computer (64.6%) was cited as the most common problem faced by students in assessing online information in campus (Table 5).

**Table 3:** Confidence in Performing Internet Related Tasks

Tasks	Perceived Self Efficacy Score	
	
	Mean	SD
1. Downloading medical books from internet	3.00	1.564

2. Search internet for classification of diseases	3.60	2.907

3. Retrieve & download full text article	3.11	1.474

**Table 4:** Use of Health Related Websites to Supplement Learning

	Total		Male		Female		P value
	
	n	%	n	%	n	%	
1. Use them now							.342

Yes	283	55.1%	122	57.5%	161	53.3%	

No	231	44.9%	90	42.5%	141	46.7%	

2. Use if had to pay							.988

Yes	126	24.6%	52	24.5%	74	24.6%	

No	387	75.4%	160	75.5%	227	75.4%	

3. Use if college pays							.937

Yes	356	69.5%	147	69.3%	209	69.7%	

No	156	30.5%	65	30.7%	91	30.3%	

**Table 5:** Distribution of Problems Faced By Students Assessing Information at Campus

Problems (not mutually inclusive)	n	%
Lack of time to use	331	64.6

Inadequate number of computers	321	62.3

Lack of support from staff	248	48.6

Other reasons	168	33.3

## Discussion

The present study examined use of internet among medical students in clinical years studying in various medical institutions in Lahore. The results of our study show that 83.97% of the students were able to use internet. A number of studies have assessed the use of internet by students in healthcare professions [6,7,14-25]. The results of our study are comparable to other studies conducted earlier in Algeria, Chile, Denmark, Finland, France, India, Jordan, Malaysia, New Zealand, Nigeria, Saudi Arabia, Sudan, Tanzania, Tunisia, Turkey, United Kingdom and the United States [6,7,14-26].

However, the frequency of internet use and its use in learning were low as compared to other studies [7,14-16,18,20,25,26]. There may be a number of reasons for this difference in use of internet. Lack of training of students as well as the various problems faced by them in utilization of internet at institution coupled with unavailability of access to subscription based online databases such as UpToDate at home where students mainly use internet may have contributed to this problem. The ability to search online databases including PubMed and PakMedinet was reported by only one third of the students in this study. This is lower as compared to the reported rates from other countries [6,7] and may be a hindrance in allowing full utilization of of internet in learning to practice evidence based medicine. Students, however, had acceptable scores in their self rated ability to perform various internet related tasks including downloading medical books from internet, searching for classification of diseases and downloading full text article. This may be attributable to the self reported nature of the rating with students over rating their perceived abilities.

A statistically significant difference in the pattern of internet access and use was seen with respect to gender and the place of internet use and ability to search various online databases for the required article. Gender difference in use of information technology has been reported previously by many studies [15,18,24,27,28]. The reason of this disparity needs to be explored since there was no statistically significant difference in frequency of internet use.

Internet provides health professionals with a cost effective medium which can be used to gain access to latest health information anytime and anywhere by anyone with resources and knowledge to make effective use of this technology. Studies have shown that online resources are not only as effective as paper based resources in answering clinical queries but are also time efficient [29]. The individuals seeking health information can even use a general search engine like Google to gain access to scholarly publication which now in fact generates more referrals than those directed by PubMed [30]. However, PubMed is more updated, gives more relevant and recent results and has online first studies in comparison to Google Scholar which rates results according to number of hits [31]. Despite all these advantages of internet, it has certain limitations which can nevertheless be rectified with proper training in accessing online content. The main problem with internet is the lack of quality control with large amounts of data of undetermined quality available. The users need to have skills to critically analyze the data and determine its accuracy, reliability and validity. This is especially important given the finding that health information seekers from general public rarely check whether the information is updated and source can be identified and is reliable [32].

The present study has certain strengths and limitations. The study provides a preliminary evaluation of the use of internet among medical students in Pakistan. It adds to the limited data on the topic from developing countries. The findings of the study can be generalized to the medical student population of the country as the study recruited participants from both public and private medical colleges having facilities and resources compared to medical institutions in the rest of the country. The study has made important points which can be elaborated in future studies. The study also has certain limitations including self reported nature of the data, differences in time available to students for internet access and utilization at different institutions and the disparities in their levels of training. In addition, analysis of technology use poses some unique challenges as it is difficult to compare results of studies conducted in different settings and time periods as many advanced tasks become simple and trivial with the passage of time.

Notwithstanding these limitations, the study provides important baseline assessment of the use of online resources and the proficiencies of students in doing so. The data from this study will be helpful in devising appropriate plans and strategies to rectify the problem of lower levels of utilization of internet and online resources by health science students in the country. It is recommended that there should be an increase in available facilities and training of students to maximize use of innumerable applications of technology in medical education and practice. The students should be trained to assess and analyze the vast amount of information available online by inclusion of basic education in utilization of information and communication technology in current curriculum. Computer assisted instruction should be introduced in medical students' training so that they do not feel handicapped later in this era of self directed learning. In this regard, strategies should be devised to involve students in their online learning. In addition, the barriers faced by students in using internet and online resources should be removed.

## Conclusions

It is concluded that the use of internet is widespread among medical students, however, its use with respect to its potential as a learning tool is less as compared to studies from other countries. Students should have skills to fully utilize online resources to improve their learning and quality of patient care bridging the gap resulting from the scarcity of resources in developing countries. Increase in awareness, availability of requisite facilities and training in computing skills are required to enable optimum utilization of digital resources by students. Further studies are needed to gather data to design cost effective ways of integrating information technology in education and training of health care professionals in developing countries.

## List of abbreviations

AIMC: Allama Iqbal Medical College, Lahore, Pakistan; FMH: Fatima Memorial Hospital College of Medicine and Dentistry, Lahore, Pakistan; MBBS: Bachelor of Medicine and Bachelor of Surgery; SMDC: Sharif Medical and Dental College, Lahore, Pakistan; SIMS: Services Institute of Medical Sciences, Lahore, Pakistan; UHS: University of Health Sciences, Lahore, Pakistan.

## Competing interests

The authors declare that they have no competing interests.

## Authors' contributions

NAJ conceived, designed and coordinated the study, participated in data acquisition, carried out analysis and interpretation, drafted the manuscript and reviewed it. MFZ and HM participated in designing the study, data collection and analysis. BAJ was involved in data input, analysis and review of manuscript. SU, MH, AR, RY and MAS were involved in data acquisition and analysis. All the authors have read and approved the final manuscript.

## Acknowledgements

We thank the students for participating in the study.

## Pre-publication history

The pre-publication history for this paper can be accessed here:

http://www.biomedcentral.com/1472-6947/11/37/prepub
